# Tumor-immune profiling of murine syngeneic tumor models as a framework to guide mechanistic studies and predict therapy response in distinct tumor microenvironments

**DOI:** 10.1371/journal.pone.0206223

**Published:** 2018-11-02

**Authors:** Jong W. Yu, Sabyasachi Bhattacharya, Niranjan Yanamandra, David Kilian, Hong Shi, Sapna Yadavilli, Yuliya Katlinskaya, Heather Kaczynski, Michael Conner, William Benson, Ashleigh Hahn, Laura Seestaller-Wehr, Meixia Bi, Nicholas J. Vitali, Lyuben Tsvetkov, Wendy Halsey, Ashley Hughes, Christopher Traini, Hui Zhou, Junping Jing, Tae Lee, David J. Figueroa, Sara Brett, Christopher B. Hopson, James F. Smothers, Axel Hoos, Roopa Srinivasan

**Affiliations:** 1 Immuno-Oncology and Combinations Research Unit, GlaxoSmithKline, Collegeville, PA, United States of America; 2 Target Sciences R&D, GlaxoSmithKline, Collegeville, PA, United States of America; 3 Oncology R&D, GlaxoSmithKline, Collegeville, PA, United States of America; Istituto Superiore di Sanità, ITALY

## Abstract

Mouse syngeneic tumor models are widely used tools to demonstrate activity of novel anti-cancer immunotherapies. Despite their widespread use, a comprehensive view of their tumor-immune compositions and their relevance to human tumors has only begun to emerge. We propose each model possesses a unique tumor-immune infiltrate profile that can be probed with immunotherapies to inform on anti-tumor mechanisms and treatment strategies in human tumors with similar profiles. In support of this endeavor, we characterized the tumor microenvironment of four commonly used models and demonstrate they encompass a range of immunogenicities, from highly immune infiltrated RENCA tumors to poorly infiltrated B16F10 tumors. Tumor cell lines for each model exhibit different intrinsic factors *in vitro* that likely influence immune infiltration upon subcutaneous implantation. Similarly, solid tumors *in vivo* for each model are unique, each enriched in distinct features ranging from pathogen response elements to antigen presentation machinery. As RENCA tumors progress in size, all major T cell populations diminish while myeloid-derived suppressor cells become more enriched, possibly driving immune suppression and tumor progression. In CT26 tumors, CD8 T cells paradoxically increase in density yet are restrained as tumor volume increases. Finally, immunotherapy treatment across these different tumor-immune landscapes segregate into responders and non-responders based on features partially dependent on pre-existing immune infiltrates. Overall, these studies provide an important resource to enhance our translation of syngeneic models to human tumors. Future mechanistic studies paired with this resource will help identify responsive patient populations and improve strategies where immunotherapies are predicted to be ineffective.

## Introduction

Therapies directly engaging the immune system have dramatically changed the clinical landscape of multiple cancers. In particular, immune checkpoint inhibitors targeting CTLA4 and PD1/PD-L1 have demonstrated significant clinical efficacy and durable responses in patients with melanoma, bladder cancer, kidney cancer, lung cancer, head and neck cancer, and Hodgkin lymphoma [1–7]. Despite this success, the majority of patients do not respond to therapy and a fraction of responders become resistant to treatment. Therefore a critical goal has been centered on understanding how the different immune composition of tumors defines responders versus non-responders and how these features can be leveraged to improve patient prognosis [8]. In support of this goal, effort has also been focused on determining whether preclinical syngeneic tumor models can represent diverse tumor-immune environments observed in humans and whether they can be probed to help stratify patients or identify novel treatments that dramatically increase clinical response [9, 10].

Over the past few decades, a large body of data classifying human tumors based on immune composition has been generated and a few major themes have emerged. First, significant heterogeneity in immune cell content is observed between cancer types and even between tumors of the same cancer type [8]. Second, despite this heterogeneity, the presence of CD8 T cells along with Th1 cells within tumors strongly correlate with improved survival for almost all cancer types observed [8]. Although all other immune cell types can be found in the tumor microenvironment, their clinical impact is either tumor type specific or the published data is inconsistent. Third, the presence of T cell attracting chemokines in the tumor microenvironment are also linked to improved immune infiltration and survival in multiple cancer types [11–15]. Fourth, tumors enriched for chemokines and infiltrated by cytotoxic T cells can be immunosuppressed by an excess of suppressive myeloid cells [16, 17] or through negative feedback mechanisms driven by IFNγ and infiltrating CD8 T cells [18]. Finally, tumors can be locked in a more severe immunosuppressed state by excluding infiltrating lymphocytes through mechanisms driven by tumor cells, cancer associated fibroblasts, suppressive myeloid cells, and the tumor vasculature [19, 20]. This state is observed across multiple cancer types, but is particularly evident in colorectal, ovarian, and pancreatic cancers [21, 22].

Recently a simple tumor classification framework was devised using T cell infiltration (TIL) status and PD-L1 expression to help stratify the complex immune environment outlined above and predict responders to immune checkpoint blockade [23, 24]. In advanced melanoma, the TIL^+^PD-L1^+^ population represents approximately 38% and these patients are proposed to respond to checkpoint blockade. In contrast, the TIL^-^PD-L1^-^ population lack immune infiltrates and are predicted to have very poor prognosis. This population is significant because it readily identifies a large proportion of patients (~41%) that will be unresponsive to conventional immunotherapy and will require alternate mechanisms to drive immune infiltration. The last major population, TIL^+^PD-L1^-^, represents ~20% of advanced melanoma patients and their tumors are predicted to be immunosuppressed by mechanisms other than checkpoint control and hence will also require novel therapies. While this framework oversimplifies the complex tumor microenvironment, it nevertheless facilitates pairing the appropriate mechanistic question to the appropriate tumor microenvironment. In a broader sense, this simple framework provides an important first step in considering patient selection criteria and rational combination strategies.

In this study, we wanted to pose a similar classification strategy over four murine syngeneic tumor models commonly used to characterize efficacy and mechanisms of cancer immunotherapies. Unlike spontaneously derived tumors, syngeneic tumors are generated by subcutaneous implantation of MHC matched tumor cell lines. Consequently, these models do not truly capture organ specific tumor microenvironments; however, within the simple framework described above each model is likely to have a unique tumor-immune infiltrate profile and collectively these models may reflect a range of tumor-immune profiles seen in humans. Therefore, baseline characterization of tumor-immune features for each model paired with therapy based mechanistic studies may inform on patient selection and combination strategies in humans with similar tumor-immune characteristics. Here, we extensively characterized immune features contributed by isolated tumor cells *in vitro* and immune features of the tumor microenvironment *in vivo* at baseline and at tumor progression. This effort will provide a foundation for future therapy based mechanistic studies to probe and predict anti-tumor responses by a given immunotherapy.

## Materials and methods

### Mouse tumor models

Female mice between six and eight weeks of age were obtained from Envigo (BALB/cAnNHsd) or Charles River Laboratory (C57BL/6NCrl). Mouse tumor cell lines RENCA (CRL-2947), B16F10 (CRL-6475), CT26 (CRL-2638), and EMT6 (CRL-2755) were obtained from American Type Culture Collection, tested for mycoplasma and other pathogens at Charles River Research Animal Diagnostics services, and cultured according to their guidelines. Low passage cells were resuspended at 1x10^6^ cells/ml in PBS for EMT6 and in a 1:1 mixture of PBS and matrigel (Corning #356231) for RENCA. B16F10 and CT26 cells were resuspended at 5x10^5^ cells/ml in PBS. 100μl of cell stock was injected subcutaneously on the shaved right flank of BALB/c mice (CT26, EMT6, and RENCA) or C57BL/6 mice (B16F10). Tumor volume growth was monitored via perpendicular tumor diameter measurements and calculated using the formula (mm^3^) = 0.52x(length) × (width)^2^. For drug treatments, mice were dosed intraperitoneal with mouse anti-mouse isotype control IgG2a (MOPC-21, Absolute AB, #T1634B05) or mouse anti-mouse OX40 (clone OX86, Absolute Ab, #AB00110-2.0). All studies were conducted in accordance with the GSK Policy on the Care, Welfare and Treatment of Laboratory Animals and were reviewed and approved by the GSK PA Institutional Animal Care and Use Committee at GSK under protocol AUP0606. Mice were housed under conditions outlined in the NIH Guide for Care and Use of Laboratory Animals in compliance with the USDA Laboratory Animal Welfare Act, in a fully accredited AAALAC facility. The animals were allowed ad libitum access to Lab Diet rodent chow and water. Mice were monitored a minimum of three times per week by the investigator or veterinary staff for clinical abnormalities which may require euthanasia. These include chronic and/or severe diarrhea leading to moderate to severe dehydration, evidence of infection that is not readily treatable, hunched posture in conjunction with other clinical signs if debilitating or prolonged for greater than three days, inability/unwillingness to ambulate to reach food or water, or other clinical signs judged by experienced technical staff to be indicative of morbidity or being in a moribund condition. Mice showing a net body weight loss >20% compared to baseline weight measurement were euthanized. Mice were euthanized in a plexiglass chamber with slow filling CO_2_ gas followed by a firm toe pinch to confirm no reflex response. An approved secondary euthanasia method of cervical dislocation was also applied.

### Tumor preparation and flow cytometry

Excised tumors were washed with PBS, dissected into smaller fragments using scalpels, and further dissociated into single cell suspensions using the Miltenyi Tumor Dissociation Kit (#130-096-730) and the GentleMACS Octo dissociator (Miltenyi #130-095-937). The digested tumors were filtered through 100μM & 30μM pre-separation filters (Miltenyi #130-098-463,130-098-458), washed with PBS, and then used for flow cytometry or RNA based analysis.

For each sample, 1 x 10^6^ cells were treated with mouse Fc Blocking solution (Miltenyi, #130-092-575) and then stained with a defined panel containing live/dead stain (Life Technologies, #L34957) and seven different labeling antibodies. Anti-CD3 (145-2C11), anti-CD4 (RM4-5), anti-CD8 (53–6.7), anti-CD45R (RA3-6B2), anti-CD25 (PC61), anti-CD11b (M1/70), anti-CD11c (HL3) were purchased from BD Biosciences. Anti-CD14 (Sa2-8), anti-FoxP3 (FJK-16s), and anti-CD45 (30-F11) were purchased from eBioscience. Anti-CD49b (DX5), anti-GR1 (RB6-8C5), and anti-F4/80 (BM8) were purchased from BioLegend. Perm/Wash Buffer (BD Biosciences, #554714) was used to permeabilize and facilitate intracellular staining. All samples were fixed with 2% paraformaldehyde, acquired on a BD FACSCanto II, and analyzed by FlowJo software (v10.1r7).

### NanoString analysis

RNA was purified from 5x10^6^ tumor cells per sample using the Qiagen RNeasy kit (#74106), QIAshredder spin columns (#79656), and DNase 1 (Qiagen#79254). For NanoString analysis, 100ng total RNA was used for hybridization per reaction using the NanoString Mouse PanCancer Immune profiling panel. The nCounter XT protocol was used for hybridization and the data was captured using a Nanostring Sprint instrument. Data analysis was performed using nSolver 2.6 and the mouse Pan Cancer Immune advanced analysis software.

### Statistical analysis

Data was analyzed using Graphpad Prism 5. For multiple group comparisons, an Analysis of Variance (ANOVA) was performed. For most data, ANOVA was performed on log10 transformed data due to unequal variances in untransformed data. The p-values for ANOVA analysis were adjusted using Tukey’s test. For analysis of survival curves, the log-rank (Mantel-Cox) test was performed to determine p-values. For Nanostring analysis, differential expression false discovery rate (FDR) was determined by the Benjamini-Yekutieli method within the nSolver 2.6 and the mouse Pan Cancer Immune advanced analysis software.

### RNA seq analysis of murine cell lines

Cell line RNA was purified using the Qiagen RNeasy kit, QIAshredder spin columns, and DNase 1. RNA quality was assessed using Agilent Bioanalyzer RNA 6000 Nano kit (#5067–1511). cDNA libraries were prepared from 1μg RNA using Illumina TruSeq RNA Library Prep mRNA kit (#RS-122-2201) and quality was confirmed using the Agilent Bioanalyzer DNA1000 Assay (#5067–1504). The libraries were quantified and normalized by qPCR using KAPA Library Quantification Kit (ABI Prism #KK4835). Library clustering was performed on a cBot with Illumina HiSeq PE Cluster kit (#PE-401-4001) and sequenced on an Illumina HiSeq1500 or HiSeq2500 to ~50 million PE 2 x 101bp reads. Fastq read data was quality checked using ArrayStudio v9 (Omicsoft). Reads were aligned to the mouse genome (NCBI37.p3/mm9) using the OSA2 algorithm and a gene model derived from NCBI RefSeq annotation release 106. Quality filters included a minimum sequencing base quality of Q20 and the exclusion of both non-paired reads and reads which map to multiple locations on the genome. Fragments per kilobase of exon per million fragments mapped (FPKM) were calculated as in [25] for the estimation of transcript abundance. The RNA-seq data was quality controlled by eliminating low-quality reads, PCR primers, adaptors, duplicates and other contaminants. Omicsoft arrayserver DNAseq analysis pipeline was employed for read mapping and variant calling. Since we also sequenced the normal tissue from appropriate mouse strains, the single nucleotide polymorphisms (SNP) identified from normal tissues were considered as germline and subtracted from SNPs identified in the cancer cell lines. We also filtered out all the variations outside of the coding region. Finally, we focused on non-synonymous point mutations and counted these mutations as the mutation load.

### IHC methods

Tumors were fixed in 10% neutral buffered formalin for 24 hours and then transferred to 70% ethanol followed by processing into paraffin blocks. The blocks were then sectioned at 4μm followed by deparaffinization and antigen retrieval in DIVA (Biocare) at 108°C for 20 minutes (except B220). IHC was then performed in the Biocare Intellipath as follows: Peroxide block for five minutes, protein block (Dako) for 10 minutes, primary antibody incubation for 45 minutes, secondary antibody incubation for 20 minutes (rabbit on rodent HRP polymer (Biocare) [CD3 (ThermoFisher, #PA1-37282), CD4 (Sino Biologicals, #50134-R001), FoxP3 (Novus, #NB100-39002), Ki67 (Biocare, #PRM325AA)] or Rat on rodent HRP polymer (Biocare) [CD8 (Affymetrix, #14–0808), F4/80 (eBioscience, #14-4081-82), B220 (eBioscience, #14-0452-85)], and Di-aminobenzidine (H_2_O_2_) (DAB) (brown chromagen) for five minutes. The sections were then counterstained with hematoxylin and scanned on a Nanozoomer (Hamamatsu) to produce digital images.

## Results

### Murine tumor cell line immune characteristics *in vitro* and tumor growth rates *in vivo*

**Fig 1:**
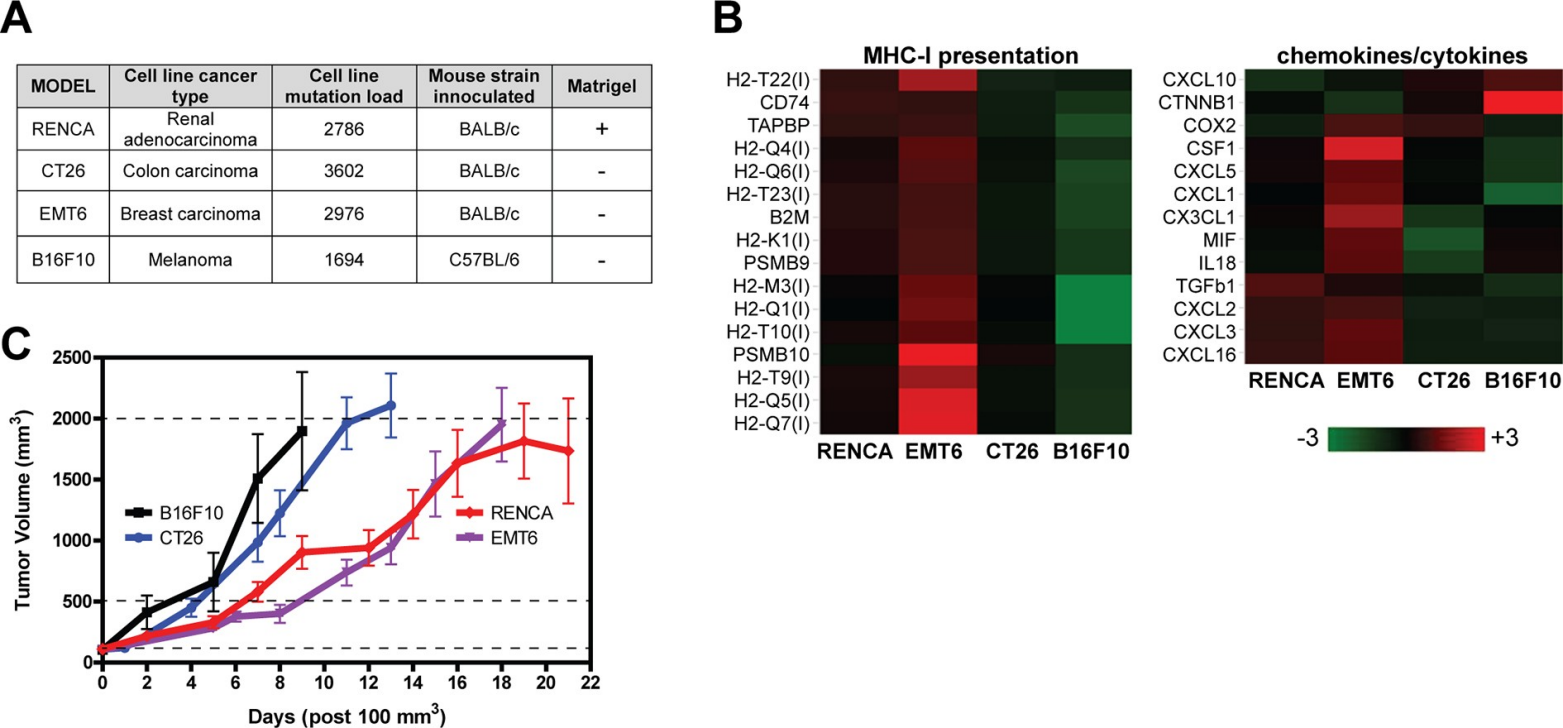
Murine tumor cell lines exhibit distinct immune characteristics *in vitro* and different growth rates post-implantation *in vivo*. (A) Murine tumor cell line background, mutational load pre-implantation, and mouse strain inoculation information. (B) RNA-seq analysis of genes involved in MHC-I presentation and chemokines/cytokines for all cell lines prior to implantation. Heatmaps were generated using log2 transformed data, with the low cutoff set at FPKM = 0.01. (I) indicates MHC-I or related genes. (C) Syngeneic tumor growth rates were assessed after reaching 100mm^3^ using 10 to 11 mice per model. Dashed lines indicate tumor collection points for three tumor sizes (100mm^3^ pretreatment, 500mm^3^, and 2000mm^3^). Error bars represent SEM.

Fig 1A outlines the murine cell lines used in this study and the mouse strains and matrices used for subcutaneous implantation. Before examining palpable tumors in mice, we assessed their pre-implantation mutational load by RNA-seq and found the CT26 cell line *in vitro* possessed the highest total non-synonymous mutation count (3,602), followed by EMT6 (2,976), RENCA (2,786) and B16F10 (1,694).

To determine immune features intrinsic to each cell line *in vitro* prior to implantation, we also examined transcript expression of immune related genes by RNA-seq. Because tumor cells can control their access to the immune system by MHC-I expression and control influx of immune cells by producing chemokines, we focused our attention to these gene sets. As shown in Fig 1B, the EMT6 cell line inherently expresses high levels of chemokines, MHC-I, and antigen presentation components as compared to all other cell lines. Of the chemokines listed, CX3CL1 and CXCL10 recruit T cells while CXCL1, CXCL2, CXCL3 and CXCL5 are involved in recruiting neutrophils or polymorphonuclear myeloid-derived suppressor cells (PMN-MDSCs). These observations suggest that upon implantation in mice, EMT6 will become tumors more highly infiltrated with T cells, neutrophils, and PMN-MDSCs when compared to RENCA, CT26, or B16F10. Our data also suggests that EMT6 tumors will be more susceptible to cytotoxic T cell responses because of higher intrinsic MHC-I expression compared to RENCA, CT26, or B16F10. The B16F10 cell line, on the other extreme, expresses very little of each, suggesting that this cell line will produce poorly immunogenic tumors upon implantation.

Using the *in vitro* RNA-seq data, we searched for additional immune differences between cell lines by identifying genes expressed three-fold higher in a given cell line over all others (S1 and S2 Tables). As described above, EMT6 expresses the highest levels of MHC-I and chemokine genes, establishing it as the most immunogenic tumor cell line. Interestingly, PD-L1, IFNGR1, and elements of B cell function (for example BAFFR and BLNK) are overexpressed in RENCA relative to the other cell lines. For B16F10, CTNNB1 (β-catenin) was significantly upregulated while almost all chemokines were suppressed (Fig 1B), indicating a possible link to melanoma intrinsic β-catenin signaling which suppresses chemokine production from tumor cells and prevents T cell infiltration [20].

Each cell line was implanted subcutaneously in the appropriate mouse strain to generate *in vivo* tumors (Fig 1A); however, RENCA was the only cell line implanted with a growth factor reduced basement membrane preparation to facilitate consistent tumor engraftment and growth kinetics. Post implantation, solid EMT6 and RENCA tumors in mice display delayed growth kinetics as compared with the rapidly growing B16F10 and CT26 tumors (Fig 1C).

### Gene expression analysis of pretreatment tumors reveals distinct classes of immunogenicity for each model

**Fig 2:**
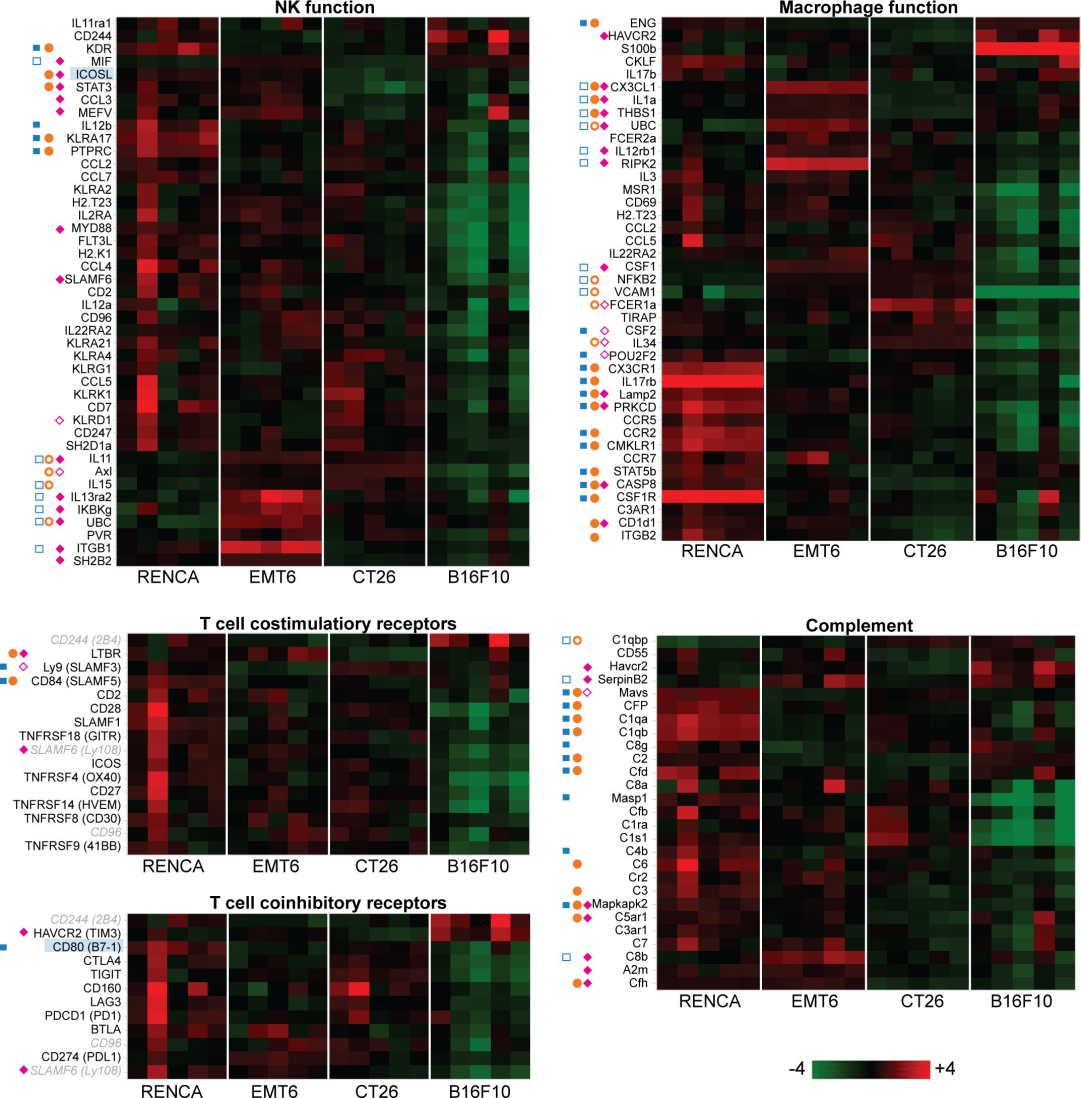
Syngeneic tumors at 100mm^3^ exhibit different immune cell and complement function profiles based on RNA expression. Heat map views of basal RNA expression levels from total tumor samples using log2 transformed data. Five tumor samples per model were analyzed. The functions of genes in grey (from the T cell costimulatory and coinhibitory receptors set) are not well defined. Genes highlighted in blue are referred to in the Results section. Filled blue squares represent transcripts from RENCA overexpressed relative to EMT6, while open blue squares represent transcripts from EMT6 overexpressed relative to RENCA (FDR < 0.1). Filled orange circles represent transcripts from RENCA overexpressed relative to CT26, while open orange circles represent transcripts from CT26 overexpressed relative to RENCA (FDR < 0.1). Filled cyan diamonds represent transcripts from EMT6 overexpressed relative to CT26, while open cyan diamonds represent transcripts from CT26 overexpressed relative to EMT6 (FDR < 0.1). Statiscially significant differences with B16F10 are not shown here.

To obtain an extensive view of immune functions within solid tumors at 100mm^3^ *in vivo* (tumor size prior to immunotherapy delivery), we examined RNA expression of over 500 immunologically relevant genes in total tumor samples inclusive of immune infiltrates using the Nanostring PanCancer Immune profiling panel. This large gene panel can be divided into specific gene sets relevant to different immune cell functions, such as NK cells, macrophages, T cells, and complement. As shown in Fig 2, RENCA tumors generally express higher levels of genes involved in each of these functions while B16F10 tumors express the least. From the standpoint of NK function and expression of T cell costimulatory and coinhibitory receptors, RENCA tumors appear to be similar to EMT6 and CT26 tumors. However, RENCA tumors differentiate from EMT6 and CT26 tumors by retaining an excess of macrophage and complement activity transcripts. This initial view suggests that each of the four syngeneic tumor models represent different tiers of tumor-immune classes (at 100mm^3^) with RENCA tumors being most infiltrated by macrophage functions and complement components and B16F10 tumors lacking all immune activities.

**Fig 3:**
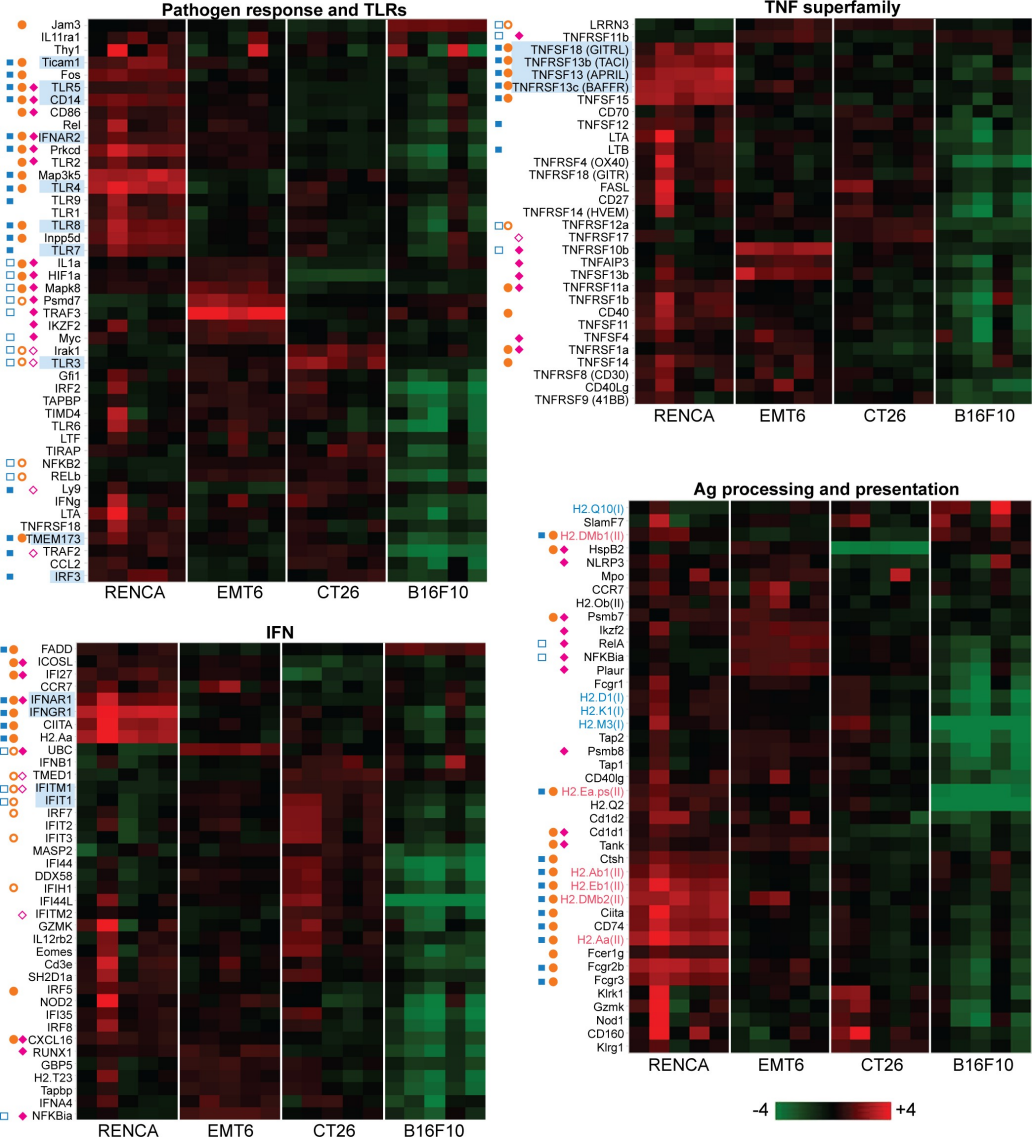
Pretreatment tumor expression profile for genes involved in innate and adaptive immune functions. Heat map views of total tumor (100mm^3^) RNA expression using log2 transformed data. Five tumor samples per model were analyzed. Genes highlighted in blue are referred to in the Results section. Filled blue squares represent transcripts from RENCA overexpressed relative to EMT6, while open blue squares represent transcripts from EMT6 overexpressed relative to RENCA (FDR < 0.1). Filled orange circles represent transcripts from RENCA overexpressed relative to CT26, while open orange circles represent transcripts from CT26 overexpressed relative to RENCA (FDR < 0.1). Filled cyan diamonds represent transcripts from EMT6 overexpressed relative to CT26, while open cyan diamonds represent transcripts from CT26 overexpressed relative to EMT6 (FDR < 0.1). Statiscially significant differences with B16F10 are not shown here.

The immune profiling panel can be further divided into gene sets for pathogen response (and TLRs), TNF and interferon signaling, and antigen processing and presentation (Fig 3). Within the pathogen response and TLR gene set, RENCA tumors are significantly enriched for innate immune responses as compared to EMT6 and CT26 tumors. For example, RENCA tumors highly express TLR4 signaling components (TLR4, CD14, and Ticam1) and TLR5, both of which are critical for NF-κB activation in response to bacterial products. RENCA tumors also highly express proteins involved in recognition of viruses and nucleic acids and the type I IFN anti-viral responses induced by TLR7, TLR8, TMEM173/STING, IRF3, IFNAR1, and IFNAR2. Given the fact that RENCA tumors are implanted with a basement membrane preparation, we cannot exclude its role in shaping the immune microenvironment as shown here. EMT6 and CT26 tumors by contrast, are uniquely enriched in proteins such as TLR3, IFIT1 and IFITM, all of which respond to or block RNA virus function.

With regard to T cell activity in tumors, MHC-II transcripts were strikingly abundant only in RENCA tumors, indicating this microenvironment is enriched with antigen presenting cells poised for T helper engagement (Fig 3). In addition, RENCA tumors are likely to be primed with T cell activity by high expression of costimulatory ligands GITRL (Fig 3), ICOSL, and CD80 (B7-1) (Fig 2) as well as elevated expression IFNα/β and IFNγ receptors (Fig 3). MHC-I expression on the other hand was consistent across all models except for B16F10, indicating that cytotoxic T cell engagement of tumors is likely to be impaired in B16F10 tumors. RENCA tumors also highly express TNF superfamily members related to B cells (TACI, APRIL, BAFFR), which may be attributed to B cell infiltration or tumor- intrinsic *in vitro* expression of B cell genes (Fig 3 and S1 Table).

**Fig 4:**
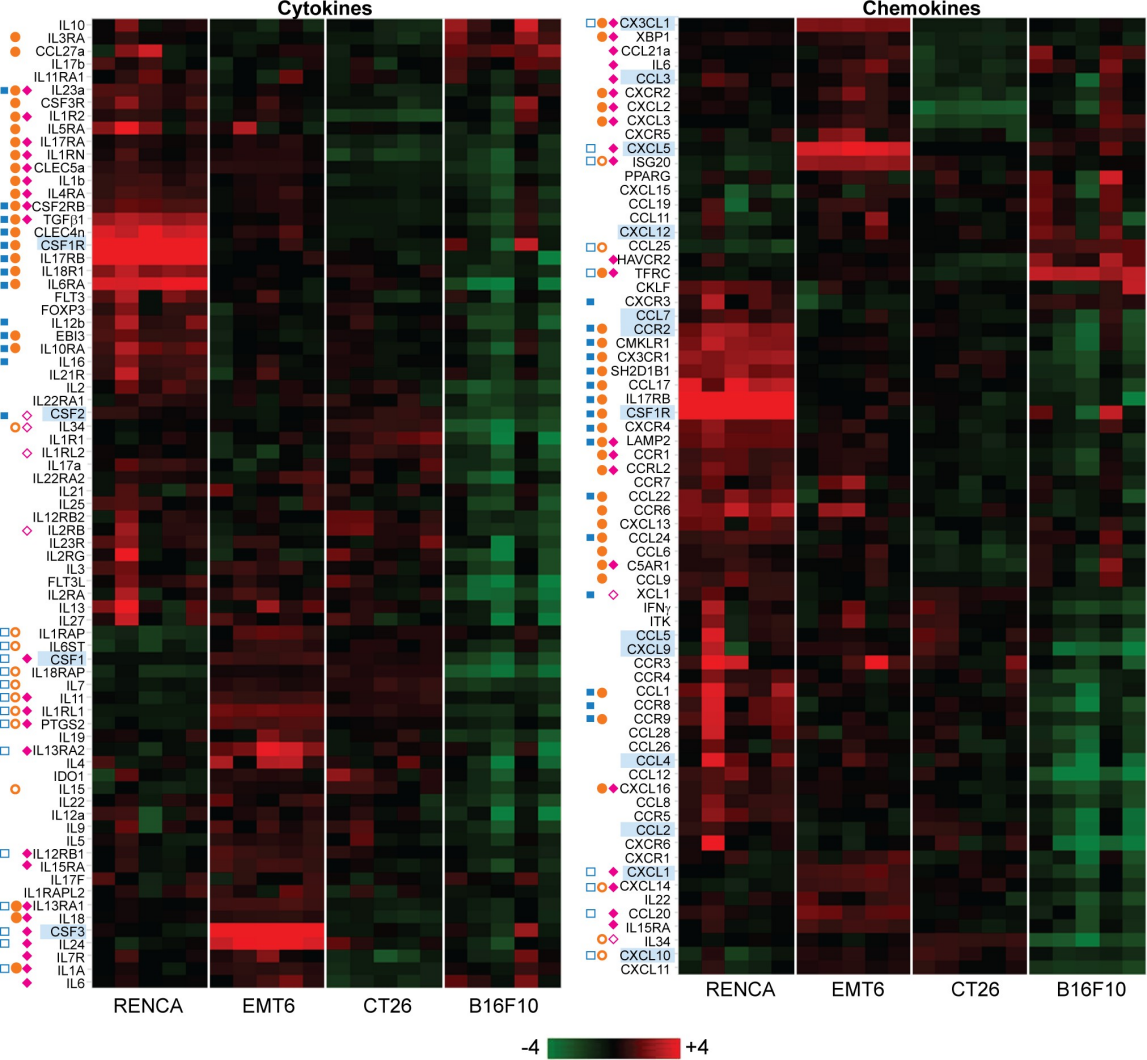
Pretreatment tumor expression profile of genes for cytokines, chemokines, and their cognate receptors. Heat map views of total tumor (100mm^3^) RNA expression using log2 transformed data. Five tumor samples per model were analyzed. Genes highlighted in blue are referred to in the Results section. Filled blue squares represent transcripts from RENCA overexpressed relative to EMT6, while open blue squares represent transcripts from EMT6 overexpressed relative to RENCA (FDR < 0.1). Filled orange circles represent transcripts from RENCA overexpressed relative to CT26, while open orange circles represent transcripts from CT26 overexpressed relative to RENCA (FDR < 0.1). Filled cyan diamonds represent transcripts from EMT6 overexpressed relative to CT26, while open cyan diamonds represent transcripts from CT26 overexpressed relative to EMT6 (FDR < 0.1). Statistically significant differences with B16F10 are not shown here.

Finally, to identify soluble factors driving the immune traits of each tumor model, we also examined transcripts for cytokines, chemokines, and their cognate receptors in the tumor microenvironment (Fig 4). Our RNA analysis revealed similar expression of key chemokines implicated in T cell attraction (CCL2, CCL3, CCL4, CCL5, CXCL9, CXCL10, and CX3CL1) [26] across the three immunogenically active models (RENCA, EMT6, and CT26). The only exceptions were CX3CL1, which is most abundant in EMT6, and CXCL10, which is predominantly expressed in both CT26 and EMT6. In addition to T cell chemoattractants, we also investigated expression of soluble factors implicated in MDSC expansion (CSF1/M-CSF, CSF2/GM-CSF, CSF3/G-CSF, and others), PMN-MDSC recruitment (CXCL1, CXCL5, CXCL6, CXCL8, and CXCL12), and monocytic MDSC recruitment (CCL2, CCL3, CCL5, CCL7, and CXCL12) [27, 28]. We found that EMT6 tumors, over all other models, expressed significantly higher levels of transcripts involved in driving MDSC expansion and PMN-MDSC infiltration (CSF1/M-CSF, CSF3/G-CSF, CXCL1, and CXCL5). This observation is consistent with our EMT6 cell line RNA analysis, which also showed significantly higher expression of CSF1 and PMN-MDSC specific chemoattractants (CXCL1 and CXCL5) when compared to RENCA, CT26, and B16F10 (Fig 1 and S1 Table). RENCA tumors by contrast express higher levels of CSF2/GM-CSF over EMT6 and higher levels of CCR2 over all other models. In this scenario, CSF2/GM-CSF may expand and elicit functional activity of CCR2^+^ monocytic-MDSCs [29–31] rather than PMN-MDSCs as seen in EMT6. Outside of MDSCs, RENCA tumors uniquely express high levels of CSF1R, which is found in numerous cell types (monocytes, DCs, neutrophils, eosinophils [32]) including immunosuppressive tumor associated macrophages (TAMs). Importantly, inhibition of CSF1R signaling can reprogram immunosuppressive TAM responses and generate anti-tumor responses in multiple mouse tumor models [33–35], and a similar approach in the RENCA model may yield similar results.

A more comprehensive view of RNA expression differences between 100mm^3^ tumors across the different models are outlined in S3–S6 Tables.

### Immune infiltrate content varies across different syngeneic models

**Fig 5:**
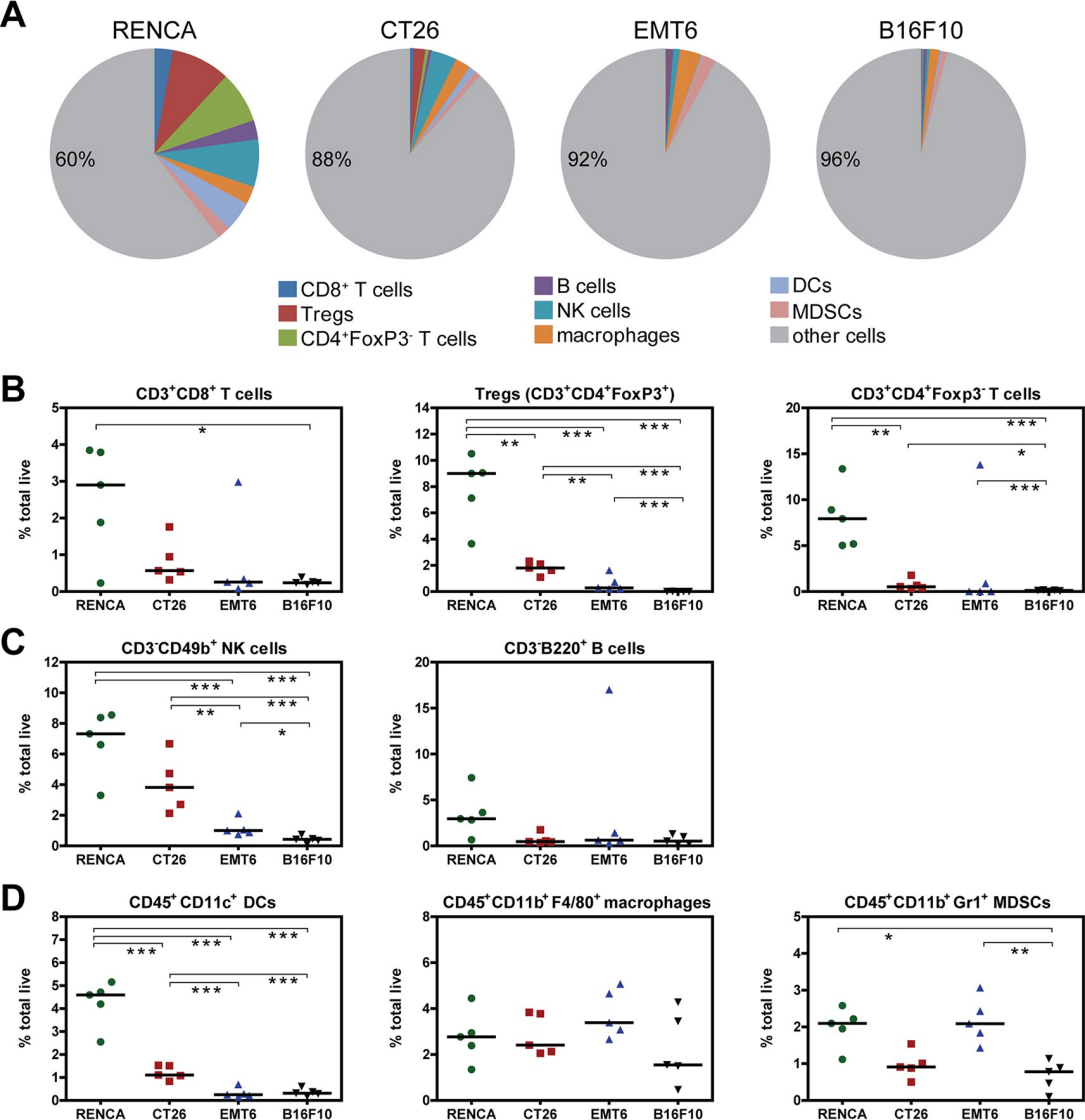
Flow cytometry analysis of key immune cell populations in 100mm^3^ tumors stratifies each syngeneic model by immune cell content. All flow cytometry data are represented as percent total live cells, which include tumor cells and immune infiltrates. (A) Pie charts summarizing the median abundance (% total live cells) of eight different immune populations in 100mm^3^ tumors from each syngeneic model. Percent values in pie charts refer to “other cells,” which includes tumor and other immune cells not captured in this analysis. Plots showing abundance of (B) T cell populations, (C) NK and B cell populations, and (D) myeloid cell populations in 100mm^3^ tumors. Medians of each immune population are indicated as bars and these values were used in the pie charts shown in (A). Statistical significance between groups: * 0.01 < p < 0.05, ** 0.001 < p < 0.01, *** p < 0.001.

To obtain a more discrete view of immune cell content within 100mm^3^ tumors, we implemented flow cytometry to assess abundance of key populations. For T cell populations examined (CD3^+^CD8^+^, CD3^+^CD4^+^Foxp3^-^(CD4^+^non-Tregs), and CD3^+^CD4^+^Foxp3^+^Tregs), RENCA tumors contained the most T cell infiltrates, followed by CT26, EMT6, and B16F10 tumors which contained the least (Fig 5A and 5B). This rank order for infiltrate abundance also holds true for NK (CD3^-^CD49b^+^) cells (Fig 5C), and dendritic cells (CD45^+^CD11c^+^) (Fig 5D). The immunosuppressive MDSC population (CD45^+^CD11b^+^Gr1^+^) (Fig 5D) is moderately elevated in RENCA and EMT6, consistent with RNA expression levels of MDSC linked cytokines, chemokines, and associated receptors in the tumor microenvironment (Fig 4). On the other hand, B cells (CD3^-^B220^+^) and macrophages (CD45^+^CD11b^+^F4/80^+^) are present at similar levels across all four tumor models (Fig 5C and 5D).

We also examined our RNA expression data to determine relative abundance of immune populations in tumors across models. Using the immune profiling analysis software (Nanostring), we observed a very similar rank order of tumor models containing most infiltrates (RENCA) to least infiltrates (B16F10) (S1 Fig). The only discrepancy between flow cytometry and RNA data was seen with macrophage abundance. The flow cytometry data indicated tumor macrophage levels were similar across all models, while the RNA data indicated macrophages were most abundant in RENCA. This inconsistency may be linked to the different methods of analysis (protein versus RNA) and different genes used to identify macrophages (FACS: CD45, CD11b, and F4/80. RNA analysis: CD58, CD84, CD163, and CYBB). Despite this difference, our data reveals a high degree of correlation between the two platforms to assess infiltration of all other immune cell types. Overall, our data identifies RENCA tumors as the most immunogenic in terms of gene signatures and immune cell content while the B16F10 tumors are the least immunogenic by every measure.

### Immune infiltrate density changes with tumor size increase for each model

**Fig 6:**
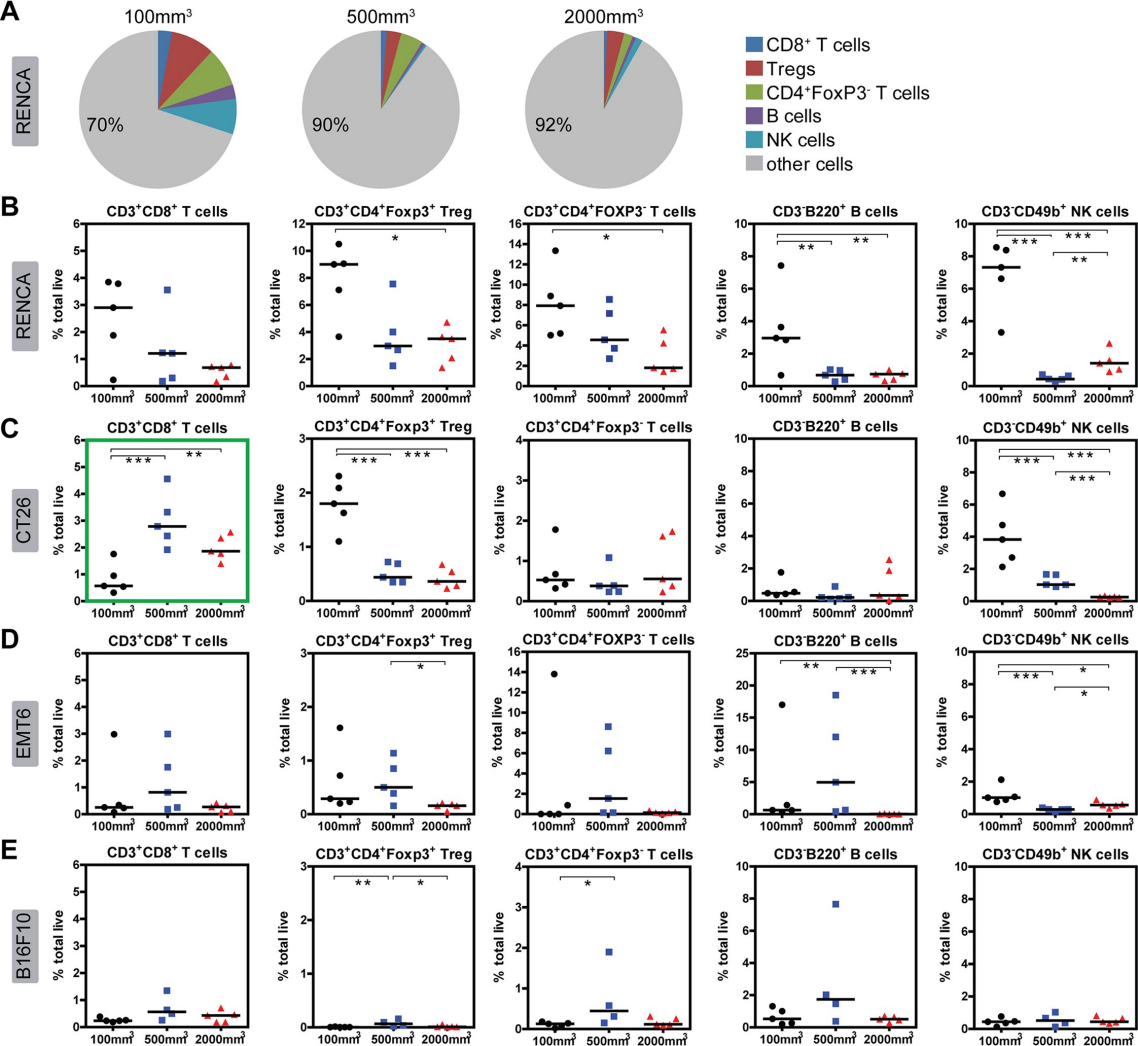
Flow cytometry analysis of lymphocyte (T/B/NK) changes within the tumor of each model as size increases. All data are represented as percent total live cells, which include tumor cells as well as immune infiltrates. (A) Pie charts summarizing the median abundance (% total live cells) of only lymphocyte (T, B, NK) populations in RENCA tumors at different sizes. Myeloid populations are excluded from this figure. Percent values in pie charts refer to “other cells,” which includes tumor and other immune cells not captured in this analysis. Plots showing abundance of T, B, and NK populations in (B) RENCA tumors, (C) CT26 tumors, (D) EMT6 tumors, and (E) B16F10 tumors. The green box highlights CD8 T cell increase with tumor volume increase in the CT26 model. Medians of each immune population are indicated as bars. Statistical significance between groups: * 0.01 < p < 0.05, ** 0.001< p < 0.01, *** p < 0.001.

**Fig 7:**
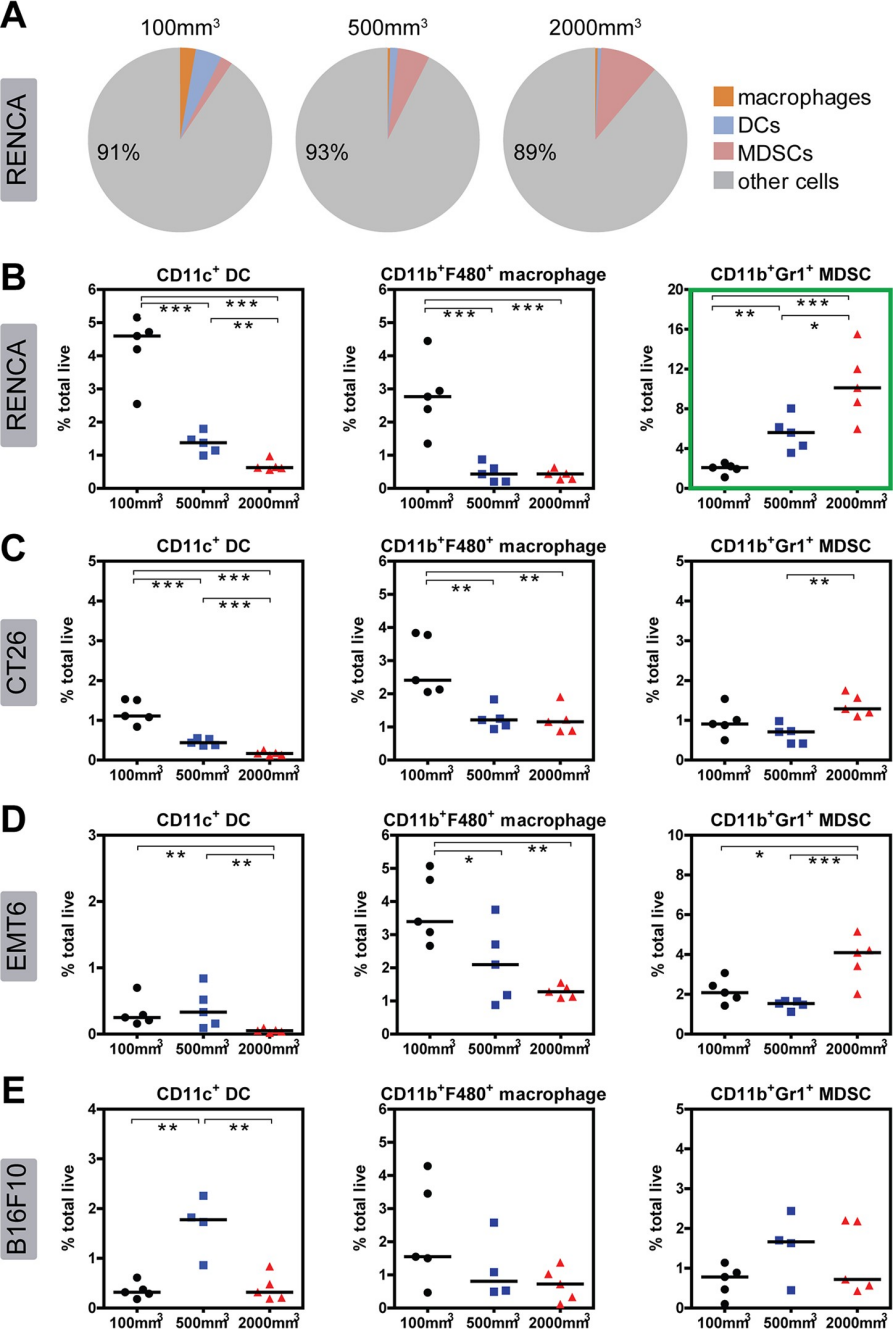
Flow cytometry analysis of myeloid population changes within the tumor of each model as size increases. All data are represented as percent total live cells, which include tumor cells as well as immune infiltrates. (A) Pie charts summarizing the median abundance (% total live cells) of myeloid populations in RENCA tumors at different sizes. Lymphocyte populations are excluded from this figure. Percent values in pie charts refer to “other cells,” which includes tumor and other immune cells not captured in this analysis. Plots showing abundance of myeloid populations in (B) RENCA tumors, (C) CT26 tumors, (D) EMT6 tumors, and (E) B16F10 tumors. The green box highlights MDSC increase with tumor volume increase in the RENCA model. Medians of each immune population are indicated as bars. Statistical significance between groups: * 0.01 < p < 0.05, ** 0.001 < p < 0.01, *** p < 0.001.

The data outlined above captures immune characteristics of all four syngeneic models at the early stage of tumor progression (prior to drug treatment). In the next set of experiments, we were interested in determining what changes occur in each model as the tumor increases in size and evades anti-tumor immunity. We first examined immune population changes within the tumor by flow cytometry. For all models, we observed a general decrease in immune cell abundance with tumor size increase. This was particularly evident in the RENCA model, which clearly shows decreases in all lymphocyte populations (CD8 T cells, Tregs, CD4^+^Foxp3^-^, B cells, and NK cells) with tumor progression (Fig 6A and 6B). Similarly the myeloid populations (DC and macrophages) in RENCA tumors also decrease in abundance with tumor size increase (Fig 7A and 7B). MDSCs were the only exception to this trend, increasing dramatically with RENCA tumor size increase (Fig 7A and 7B). This observation suggests that despite very high levels of immune infiltrates at 100mm^3^, anti-tumor immunity may be blunted by the expanding MDSC population in the RENCA model.

CT26 tumors follow a similar trend to RENCA, with most immune populations decreasing as a proportion of total cells as tumor size increases (Figs 6C and 7C). These include Tregs, NK cells, DCs, and macrophages. Unique to CT26, CD8 T cells surprisingly increase in frequency as the tumor expands and evades the immune system (Fig 6C). For EMT6 tumors, all T cell populations remain relatively low and unchanged in abundance with tumor size progression (Fig 6D). EMT6 tumors exhibit a measurable and significant decrease in B and NK cells (Fig 6D) as well as DC and macrophages (Fig 7D). Moreover, EMT6 tumors also exhibit a modest increase in MDSCs as tumor size increases (Fig 7D). For B16F10 tumors, immune populations in tumors are inherently low in abundance at 100mm^3^ and remain low as tumor size increases (Figs 6E and 7E).

Along with flow cytometry data, we also examined cell abundance changes during tumor progression using our RNA expression data (S2 Fig) and again observed reasonable correlation between the two different platforms.

### Immune infiltrates are spatially confined to the invasive margin of tumors during tumor progression

**Fig 8:**
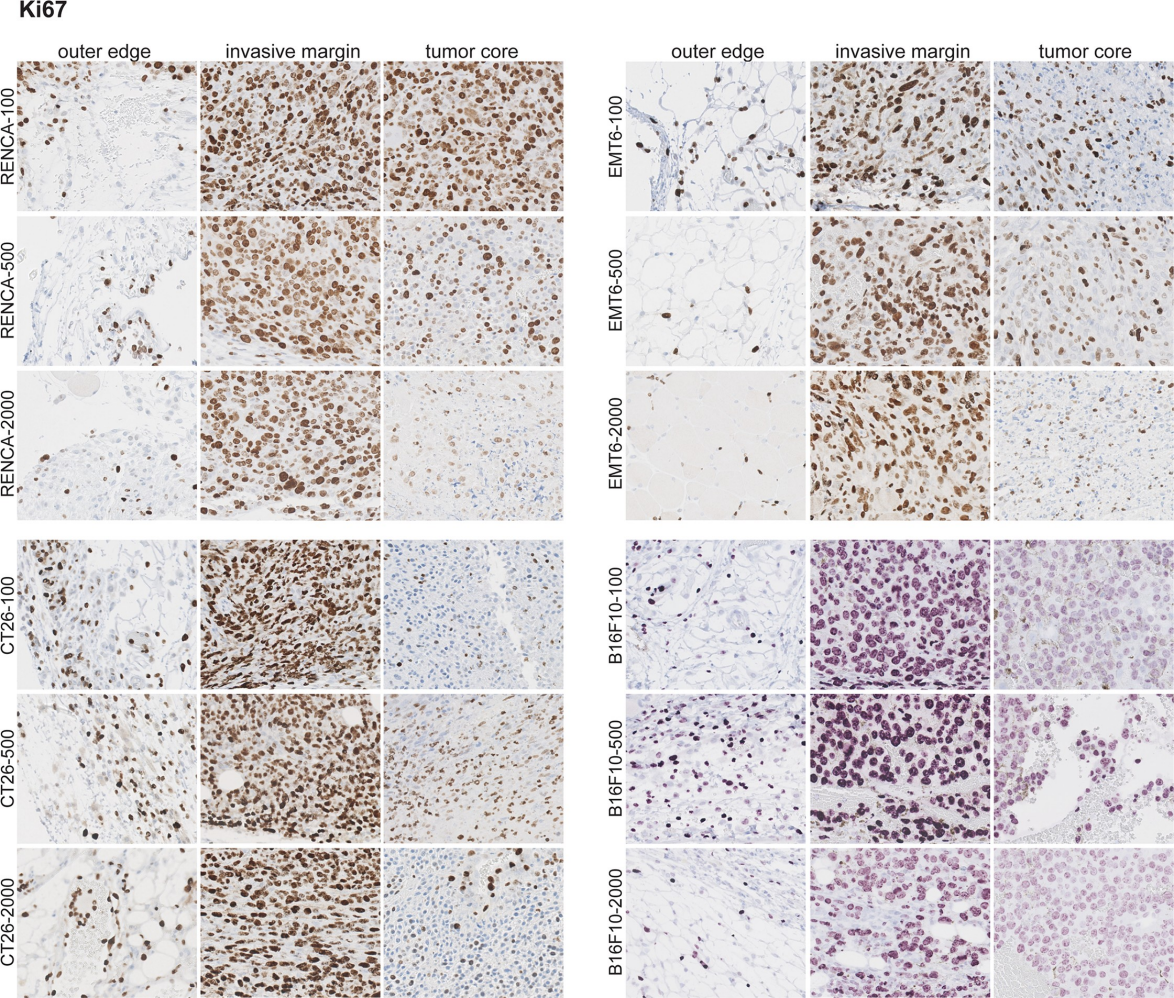
Ki67 staining of tumor samples reveals proliferation at the invasive margin. IHC was performed on fixed and paraffin embedded tumor samples across the different models and across all tumor sizes. Five mice per model at each tumor size were used for this analysis. A representative image for each is shown.

Although flow cytometry and RNA data provide information on relative abundance of immune infiltrates, these methods do not provide insight on their spatial location within the tumor. We therefore performed immunohistochemistry on fixed and paraffin embedded tumor sections from all four models across all tumor progression sizes. We stained for CD3, B220, F4/80, and Ki67 and assessed localization outside the tumor (outer edge), the tumor rim (invasive margin), and the tumor core. Examination of the proliferation marker Ki67 revealed that tumors from all models and all sizes exhibit robust proliferation at the invasive margin (Fig 8). The tumor core by contrast is generally nonproliferative in all models across all tumor sizes, with the only exception being RENCA tumors at 100mm^3^. This is consistent with the properties of subcutaneous syngeneic models, where tumors rapidly grow at the edges and outcompete for nutrients needed at the core.

**Fig 9:**
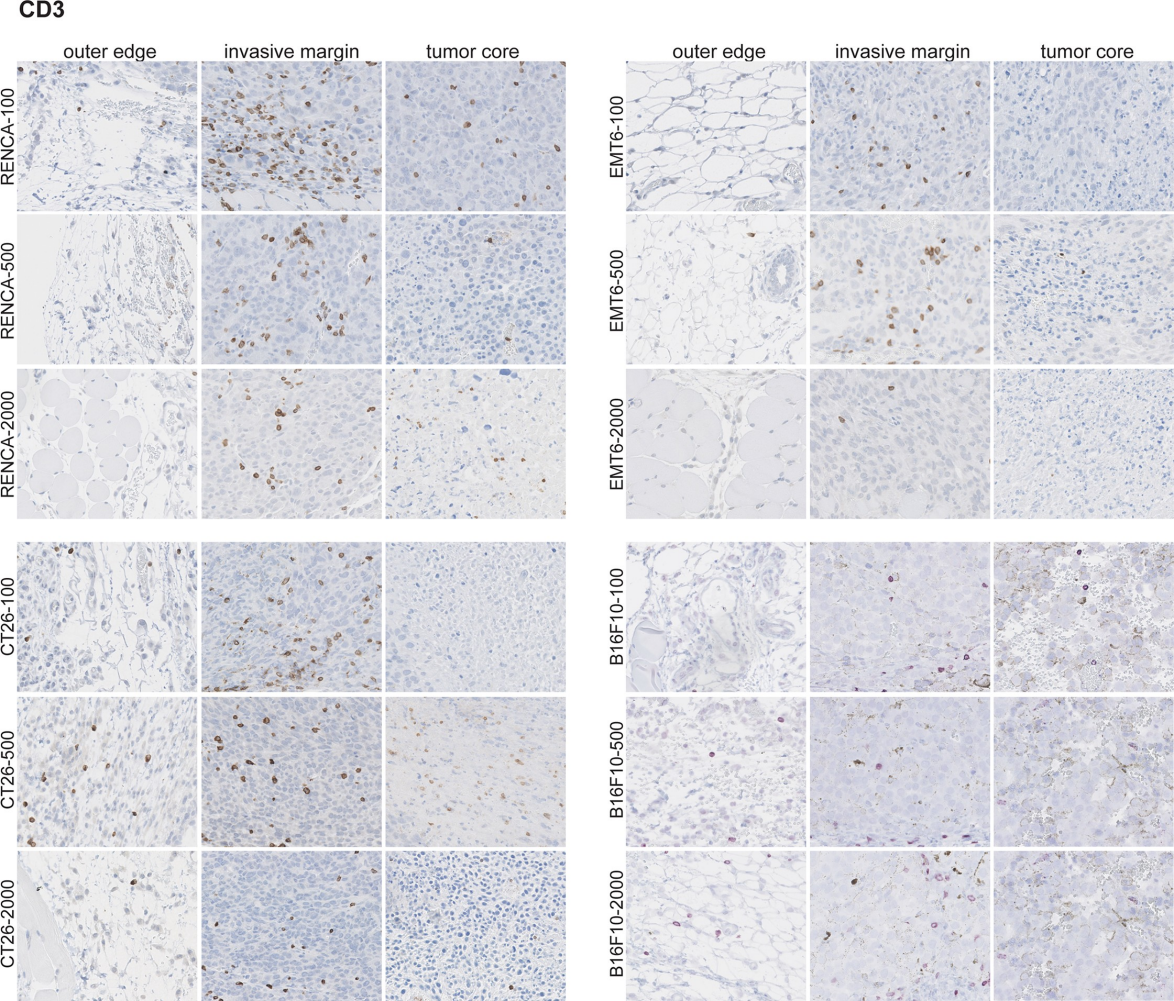
CD3^+^ cells are confined predominantly to the invasive margin in untreated tumors. IHC was performed on fixed and paraffin embedded tumor samples across the different models and across all tumor sizes. Five mice per model at each tumor size were used for this analysis. A representative image for each is shown.

For all tumor models, CD3^+^ T cells are most prevalent in the invasive margin, with little present in the outer edge or tumor core (Fig 9). As tumors increase in size, the relative density of CD3^+^ T cells in the tumor leading edge qualitatively decreases (particularly in RENCA). Similar to CD3^+^ staining, markers for all other cell types (especially with F4/80^+^ and B220^+^) also stain almost exclusively in the invasive margin (S3–S4 Figs). It is important to note in human tumors, myeloid and all T cell populations are present in the invasive margin and as well as the core of multiple tumor types [8]. This difference in spatial location of immune cells points to possible limitations or caveats of mouse syngeneic models which may limit the translatability to human tumors [36]. For example, syngeneic tumors are generated by subcutaneous injection of an established tumor cell line which do not have the opportunity to progress in typical fashion from initiation to fully formed tumors in the appropriate organ site. Moreover, syngeneic tumor growth is also extremely rapid compared to human tumors, therefore immune system exposure will be rapid and this in turn may skew the spatial distribution of immune infiltrates that develops over typical tumor progression.

### Tumor-immune function changes occur as tumors progress in size across models

To obtain a mechanistic view of immune changes occurring with tumor progression, we probed our tumor RNA data for genes that were significantly up or down regulated (FDR < 0.1) in 2000mm^3^ versus 100mm^3^ tumors (S7–S9 Tables). We first focused our attention to a list of 18 genes directly linked to or implicated in immune suppression within the tumor. These genes can be categorized in five groups, which include: immunosuppressive cytokines (IL10 and TGFβ), immunosuppressive Tregs and myeloid populations (FOXP3, CCR2, CSF1R, CSF2/GMCSF, and CXCL12), nutrient depleting and redox enzymes (IDO, ARG1, and NOS2/iNOS), T cell inhibitory ligands and receptors (CD274/PD-L1, Pdcd1lg2/PD-L2, CTLA4, and HAVCR2/TIM3), and tumor vasculature and extracellular matrix elements (VEGFA, AXL, FN1/fibronectin, and Col1a1/collagen). In this analysis, we were not able to capture tumor intrinsic immune suppression by β-catenin signaling (Fig 1B) because of our limited probeset.

Our analysis revealed that as RENCA tumors progress, they surprisingly down regulate multiple immunosuppressive elements outlined above, including IL10, FOXP3, CTLA4, and others. However we also observed increases in NOS2/iNOS and TGFβ2 transcripts, key drivers of MDSC mediated immune suppression [27] (S7 Table). In this example, the statistical significance for NOS2 increase did not meet our stringent cutoff of FDR < 0.1 (p = 0.035, FDR = 0.354), nevertheless NOS2 was upregulated only in progressing RENCA tumors, not in CT26 or EMT6 tumors. Along with NOS2 (specifically linked to monocytic MDSC suppressive activity [37]), we also observed significant upregulation of MIF, a multifunctional protein that possesses isomerase activity as well as pro-monocytic MDSC activity in humans and mice [38, 39]. These findings are consistent with our observation that MDSC density increases as RENCA tumors progress in size (Fig 7A and 7B) and supports a role for monocytic MDSCs in driving tumor progression. In addition to MDSCs, late stage RENCA tumors also generate excess VEGFA which may impact the tumor vasculature and contribute to tumor immunosuppression (S7 Table). High VEGF expression has been linked to decreased T cell infiltration in ovarian cancers [22] and has been demonstrated to block Th1 and CD8 T cell function [40] and block dendritic cell maturation [41, 42]. Moreover, anti-VEGF therapy appears to improve T cell infiltration in some preclinical tumor models [43, 44].

In progressing CT26 tumors, a couple of interesting genes related to the extracellular matrix (ECM) are upregulated (S8 Table). These include the ECM component fibronectin and AXL, a receptor tyrosine kinase that transmits signals from the ECM to regulate adhesion, migration, and survival of cancer cells. One possible mechanism of immunosuppression occurs by physically blocking T cell transit into the tumor. Fibronectin and collagen can be produced by tumor cells or cancer associated fibroblasts and a recent study has shown that dense regions of both can profoundly inhibit T cell migration [45]. In fact, collagen engagement of collagen receptors on lymphocytes can also block migration and inhibit immune activity [46, 47]. Although our data shows that T cells can still accumulate in progressing CT26 tumors (Fig 6C), fibronectin may still affect the mobility and cytotoxicity of infiltrated T cells. AXL, on the other hand, is an interesting ECM sensing receptor involved in both tumor cell metastasis and immune cell function. Current evidence suggests that inhibition of AXL may enhance T cell infiltration and function with radiation therapy [48]. Moreover, high levels of AXL have been linked to resistance of anti-PD1 therapy in humans [49].

In EMT6 tumors, we also detected groups of genes that were up and down regulated with tumor size increase, but we did not observe expression patterns that point towards immune silencing and tumor progression (S9 Table). As for B16F10 tumors, we were not able to detect statistically significant expression differences between 2000mm^3^ tumors and 100mm^3^ tumors.

### Immunotherapy treatment promotes survival in different syngeneic models

**Fig 10:**
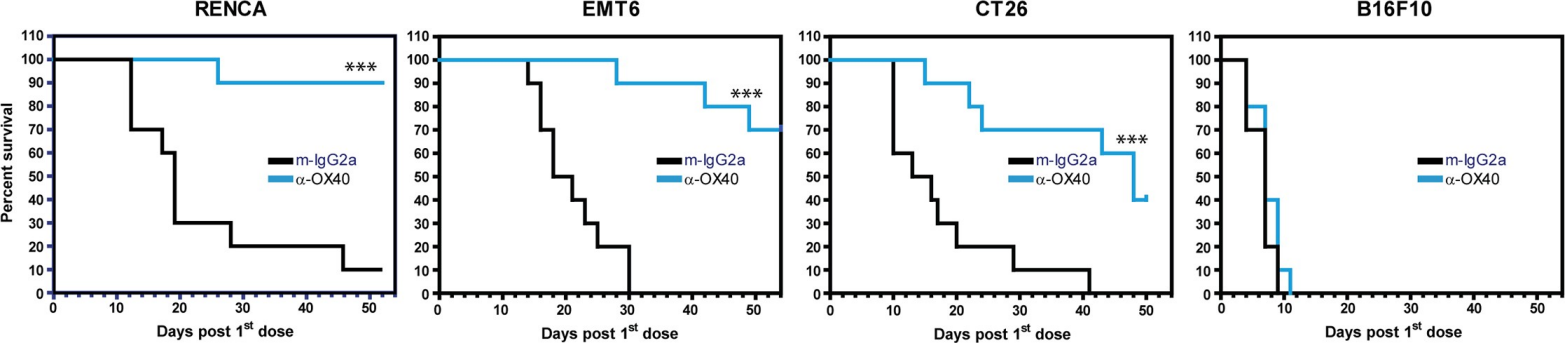
Immunotherapy treatment enhances survival in models with distinct tumor microenvironments. For each model, mice were dosed with either 1μg α-OX40 antibody or 400μg isotype control antibody every three or four days (six doses in total). Ten mice were used per treatment group. Mice were categorized as dead when the tumor volume reached 2000mm^3^. Statistical significance between groups: * 0.01 < p < 0.05, ** 0.001 < p < 0.01, *** p < 0.001.

To support our ongoing clinical trial evaluating an anti-OX40 antibody, we next asked if treatment with this T cell costimulatory receptor agonist (also capable of depleting OX40 enriched cells through Fc receptor interactions) induces different responses in these four different tumor microenvironments. Fig 10 shows that six doses of anti-OX40 treatment over three weeks effectively promotes survival in all tumor models except B16F10. Across the three responsive tumor models, anti-OX40 treatment was most effective in the RENCA model and moderately effective in the CT26 model. Given our observation that B16F10 are the least immunogenic tumors, we were not surprised by the ineffectiveness of the anti-OX40 treatment. Overall, this data demonstrates that a given immunotherapy exhibits varying anti-tumor responses in distinct tumor-immune microenvironments.

## Discussion

In this report, we present a detailed characterization of four commonly used mouse syngeneic tumor models. This endeavor is largely descriptive and is focused primarily on understanding model differences within the tumor-immune microenvironment and framing these features to clinically relevant tumor-immune categories. However, there are some factors limiting translatability of these models to human tumors that must be considered when examining our data. First, syngeneic tumors are not generated spontaneously, but rather by subcutaneous implantation of a given MHC matched cell line derived from established and fully developed tumors. Second, tumor cell lines used in syngeneic models originate from a range of tissue types or organs surrounded by a microenvironment that is distinct from the site of subcutaneous implantation. Finally, these implanted tumors progress much more rapidly than typical spontaneous tumors. These observations highlight the fact that syngeneic tumor models do not truly reflect typical tumor progression in relevant organ specific environments. In this regard, genetically engineered mouse (GEM) models of cancer (germline or conditional) are more physiologically relevant as they recapitulate the appropriate kinetics and stepwise progression from tumor initiation to tumor establishment at the site of tumor origin [50]. This difference in tumor initiation and location of tumor growth between subcutaneous syngeneic versus GEM models likely engenders different inherent immune infiltration profiles and in turn different anti-tumor immune responses. For example, spontaneous lung or pancreatic tumors from GEM models elicit weak in tumor T cell responses that diminishes over time, whereas subcutaneously implanted tumors derived from cell lines of the same spontaneous tumors induce significantly greater T cell infiltration and anti-tumor responses [51, 52].

The stark contrast between syngeneic tumor models and GEM models highlights the importance in framing syngeneic tumor model data in the appropriate context. Our focus is strictly centered on characterizing the tumor-immune infiltrate profile of each syngeneic model, and collectively using a panel of tumor models with different profiles as a framework to understand how these immune infiltrate differences translate to anti-tumor responses. Our larger and long term goal is to enhance our translation of mouse model studies in identifying immunotherapy responsive patients based on their tumor-immune profile and improving treatment strategies where immunotherapies are predicted to be ineffective.

Our studies reveal that four routinely used models phenotypically cover a broad range of tumor-immunogenicities, from immune rich RENCA tumors to immune poor B16F10 tumors. RENCA tumors, when analyzed at our typical pretreatment size (100mm^3^), are the most concentrated in immune cells and immune function. Flow cytometry and transcript data show that RENCA tumors contain the highest density of all immune cell types analyzed, with few exceptions. RENCA tumors are also highly enriched for complement activity, bacterial product recognition (via TLR4 and TLR5), as well as viral/nucleic acid recognition (TLR7, TLR8, TMEM173/STING). Moreover, these tumors are also highly enriched in factors that recruit or support suppressive monocytic MDSC or tumor associated macrophage populations (CCR2, CSF1R, and GM-CSF/CSF2). EMT6 tumors, by contrast, are not as immune infiltrated as RENCA, yet they are uniquely enriched in factors that support PMN-MDSC suppressive activity.

One question that emerges from these observations is—what determines the level of infiltrate and immune functions in these tumors? To some degree, tumor cell intrinsic factors may play a role. Interestingly our *in vitro* cell line analysis predicted EMT6 would be the most immunogenic while the B16F10 model would be the least based on their intrinsic MHC-I and chemokine expression levels. Although our analysis show that EMT6 tumors are immune infiltrated, RENCA tumors are clearly the most immunogenic as described above, and it is likely other factors such as oncogenic signaling pathways or stromal components are contributing to the tumor immune content [20, 53, 54]. From a technical standpoint, we also cannot rule out the possibility that implantation with a basement membrane preparation may artificially retain immune cells within solid RENCA tumors. With B16F10 tumors, oncogenic β-catenin signaling in melanoma cells may create an environment devoid of immune infiltrates by suppressing tumor intrinsic chemokine production [20]. Another tumor intrinsic factor driving immune infiltration is tumor mutational load, and this feature has been linked to PD-L1 expression in human tumors [55], immune infiltrates [56, 57], and most importantly clinical response to checkpoint inhibitors [58–60]. Our mouse models loosely align with this trend, as the models with detectable immune infiltrates and PD-L1 expression (RENCA, EMT6, and CT26) have higher non-synonymous mutation counts while the poorly infiltrated B16F10 model has the least tumor mutations.

Along with tumor cell intrinsic factors, tumor cell extrinsic factors can also determine the level of immune infiltrate and function in tumors. One parameter of interest is the inherent immune biases of the inbred mouse strains C57BL/6 (which harbor B16F10 tumors) and BALB/c (which harbor RENCA, CT26, and EMT6 tumors). In C57BL/6 mice, T cells produce significant levels of IFNγ and macrophages produce significant levels of TNFα and IL12 upon activation, while activated T cells and macrophages from BALB/c mice produce much less of the same respective cytokines [61–63]. Despite the strong Th1 bias of C57BL/6 mice, our data indicates that B16F10 tumors in the same strain are devoid of immune infiltrates, whereas tumors implanted in Th2 biased BALB/c mice are immune infiltrated to a much greater extent. This observation suggests that in mice, strain specific immune biases that support or suppress T cell immunity cannot override tumor cell intrinsic or other extrinsic factors that dictate tumor immune infiltrate levels. Another extrinsic parameter that likely contributes to the tumor-immune phenotype is the organ specific microenvironment surrounding the tumor. This compartment consists of multiple components (including organ specific immune cells, circulating immune cells, endothelial cells, fibroblasts, extracellular matrix proteins, and others), and these elements can uniquely shape tumor proliferation, vascularization, metastasis, as well as tumor-immune infiltration [64]. In support of this view, previous studies have shown that orthotopic implantation of RENCA or CT26 yield a more immunosuppressed tumor-immune infiltrate profile that is not as responsive to immunotherapy when compared to immunotherapy responsive subcutaneously implanted tumors [65]. As described above, spontaneous lung or pancreatic tumors from GEM models also induce weak intratumoral T cell responses, whereas subcutaneously implanted tumors derived from cell lines of the same tumors induce greater T cell infiltration and anti-tumor responses [51, 52].

Another mechanistic question emerging from our study centers on how different tumor environments evade immunosurveillance as tumors progress. By tracking immune population and gene expression changes with increasing tumor size, we were able to uncover a number of mechanisms unique to each model. From a general standpoint, all immune infiltrated models (RENCA, EMT6, and CT26) exhibit immune population loss with increasing tumor size, suggesting that the tumor can outgrow the infiltrates or prevent further influx of immune cells. Yet for RENCA, tumor progression also yielded an overall increase in suppressive myeloid cell (MDSC) density, as well as increased expression of genes that drive monocytic MDSC immunosuppression (NOS2/iNOS, MIF, and TGFβ2) [27, 37–39]. In human cancers, the correlation between myeloid-inflamed tumors with immunosuppression and poor prognosis has been noted for some time [17] and recent data further establishes this correlation to head and neck squamous cell carcinomas and pancreatic ductal adenocarcinomas [16]. As for CT26 tumors, CD8 T cells paradoxically increase in relative numbers as the tumor volume increases. It is not clear how the CD8 T cells are turned off, given the fact that obvious suppressive mechanisms (such as Tregs or suppressive myeloid cells) are not concomitantly upregulated. However, one possible mechanism, as outlined in the results section, may occur though extracellular matrix driven dampening of adaptive immunity. Another possible restraining mechanism is T cell exhaustion, which is driven by prolonged T cell exposure to antigen leading to progressive loss of function [66]. A hallmark of exhausted CD8 T cells is sustained upregulation of multiple inhibitory receptors including PD1/PDCD1, 2B4/CD244, KLRA7/Ly49D-GE, KLRA3/Ly49c, CD160, LAG-3, CTLA4, and others [67]. Although our gene expression analysis of progressing CT26 tumors revealed an increase in CD8 T cell transcripts, increases in inhibitory receptor transcripts were not detected. While this observation is not conclusive, this finding and published data indicating that anti-PD1 monotherapy is not effective in treating CT26 tumors [68, 69] suggest that T cell dampening in CT26 may extend beyond PD1 driven exhaustion. It is important to note that the mechanisms proposed here are based on a focused set of genes known to impact anti-tumor immunity. Additional analysis is needed to assess whether our larger data set identifies other mechanisms that impact evasion of anti-tumor immunity.

Perhaps the most critical aspect of this work lies in our ability to position these models in a simple framework to guide patient population selection and combination strategies for a given immunotherapy. Despite the limitations of syngeneic models, we propose the different tumor-immune microenvironments observed in each model collectively reflect a range of tumor-immune infiltrate profiles in humans. We also propose that probing a wide panel of syngeneic models and their unique tumor microenvironments with a given immunotherapy will yield insights into features (such as TIL^+^PD-L1^+^ for anti-PD1) that define responding versus non-responding tumors in humans. Our baseline model characterization reveals that EMT6, CT26, and RENCA are immune infiltrated with each model possessing unique features. Across all models, anti-OX40 treatment is most effective in promoting survival in RENCA, moderately effective in CT26, and ineffective in the poorly immunogenic B16F10. Future studies examining immune changes *in vivo* with drug treatment will be instrumental in identifying components that drive therapy response and identifying patient selection criteria for a given drug. As we expand our studies toward therapy based mechanistic changes in these models, we plan to connect the descriptive data generated here with drug induced immune changes to define a more robust tumor signature that will identify patients responsive to anti-OX40 therapy as a monotherapy or in combination with other anticancer drugs.

## Supporting information

S1 Table Murine cell line RNA-seq data (in FPKM) identifying immune genes expressed three-fold higher in a given cell line (in red) above all other cell lines. This comparison is focused on a list of 669 mouse immunology genes as defined by Nanostring. Transcripts with FPKM values <10 across all four models were removed from this analysis. Transcripts that do not adhere to the criteria of being three-fold higher in a given cell line above all other cell lines were also removed.
(XLSX)

S2 Table Murine cell line RNA-seq data (in FPKM) identifying all genes expressed three-fold higher in a given cell line (in red) above all other cell lines. Comprehensive list covering all transcripts, including immunology defined genes listed in S1 Table. Transcripts with FPKM values <10 across all four models were removed from this analysis. Transcripts that do not adhere to the criteria of being three-fold higher in a given cell line above all other cell lines were also removed.
(XLSX)

S3 Table Differentially expressed genes in pretreatment EMT6 tumors versus RENCA tumors. EMT6 tumor (100mm^3^) transcripts upregulated or downregulated relative to RENCA tumors (100mm^3^) with FDR < 0.1. Differential expression determined within the Nanostring PanCancer Immune profiling panel.
(XLSX)

S4 Table Differentially expressed genes in pretreatment CT26 tumors versus RENCA tumors. CT26 tumor (100mm^3^) transcripts upregulated or downregulated relative to RENCA tumors (100mm^3^) with FDR < 0.1. Differential expression determined within the Nanostring PanCancer Immune profiling panel.
(XLSX)

S5 Table Differentially expressed genes in pretreatment B16F10 tumors versus RENCA tumors. B16F10 tumor (100mm^3^) transcripts upregulated or downregulated relative to RENCA tumors (100mm^3^) with FDR < 0.1. Differential expression determined within the Nanostring PanCancer Immune profiling panel.
(XLSX)

S6 Table Differentially expressed genes in pretreatment EMT6 tumors versus CT26 tumors. EMT6 tumor (100mm^3^) transcripts upregulated or downregulated relative to CT26 tumors (100mm^3^) with FDR < 0.1. Differential expression determined within the Nanostring PanCancer Immune profiling panel.
(XLSX)

S7 Table Gene expression changes comparing 2000mm^3^ versus 100mm^3^ RENCA tumors. Transcripts differentially expressed with FDR < 0.1 are listed. Differential expression determined within the Nanostring PanCancer Immune profiling panel.
(XLSX)

S8 Table Gene expression changes comparing 2000mm^3^ versus 100mm^3^ CT26 tumors. Transcripts differentially expressed with FDR < 0.1 are listed. Differential expression determined within the Nanostring PanCancer Immune profiling panel.
(XLSX)

S9 Table Gene expression changes comparing 2000mm^3^ versus 100mm^3^ EMT6 tumors. Transcripts differentially expressed with FDR < 0.1 are listed. Differential expression determined within the Nanostring PanCancer Immune profiling panel.
(XLSX)

S1 Fig RNA analysis of key immune cell populations in 100mm^3^ tumors across different models. Abundance of immune cell populations was determined by total tumor RNA analysis using the PanCancer Immune profiling panel. Cell type expression scores are expressed in log scale and comparative flow cytometry data is identical to Fig 5. (A) T cell populations. (B) NK, B, and myeloid cell populations. The p-values listed at the top of each graph reflect correlation and consistency of expression data with the cell specific gene signature. For p-values > 0.05, we cross compared with FACS data and found correlation between both platforms. Data with p > 0.05 should be taken as a preliminary guide in the absence of FACS data. For cell types without p-values, only one gene was used to estimate population abundance. Medians of each immune population are indicated as bars. Statistical significance between groups: * 0.01 < p < 0.05, ** 0.001 < p < 0.01, *** p < 0.001.
(TIF)

S2 Fig RNA analysis of immune cell population changes within the tumor as size increases. Abundance of immune cell populations was determined by total tumor RNA analysis using the PanCancer Immune profiling panel. Immune populations changes with tumor progression in (A) RENCA, (B) CT26, (C) EMT6, and (D) B16F10. The p-values listed at the top of each graph reflect correlation and consistency of expression data with the cell specific gene signature. Data with p > 0.05 should be taken as a preliminary guide in the absence of FACS data. For cell types without p-values, only one gene was used to estimate population abundance. The green box highlights CD8 T cell increase with tumor volume increase in the CT26 model, which is consistent with FACS data. Medians of each immune population are indicated as bars. Statistical significance between groups: * 0.01 < p < 0.05, ** 0.001 < p < 0.01, *** p < 0.001.
(TIF)

S3 Fig F4/80^+^ cells are confined predominantly to the invasive margin in untreated tumors. IHC was performed on fixed and paraffin embedded tumor samples across the different models and across all tumor sizes. Five mice per model at each tumor size were used for this analysis. A representative image for each is shown.
(TIF)

S4 Fig B220^+^ cells are confined predominantly to the invasive margin in untreated tumors. IHC was performed on fixed and paraffin embedded tumor samples across the different models and across all tumor sizes. Five mice per model at each tumor size were used for this analysis. A representative image for each is shown.
(TIF)
