# Supplementary figures and images for: Tumor-immune profiling of murine syngeneic tumor models as a framework to guide mechanistic studies and predict therapy response in distinct tumor microenvironments
Source: PLoS One. 2018 Nov 2;13(11):e0206223. doi: 10.1371/journal.pone.0206223 (PMC6214511; doi:10.1371/journal.pone.0206223)

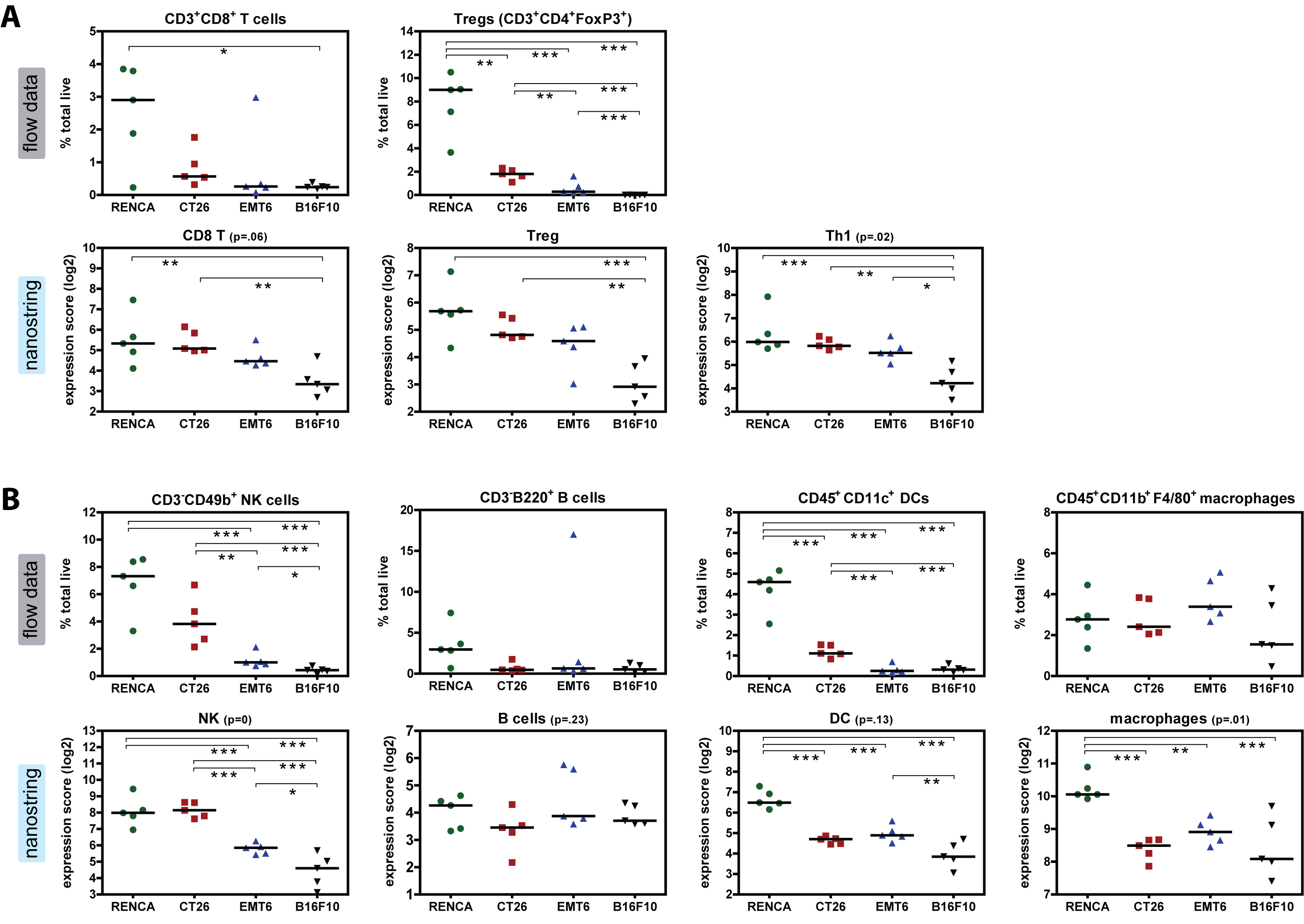

Supplement: S1 Fig — Abundance of immune cell populations was determined by total tumor RNA analysis using the PanCancer Immune profiling panel. Cell type expression scores are expressed in log scale and comparative flow cytometry data is identical to Fig 5. (A) T cell populations. (B) NK, B, and myeloid cell populations. The p-values listed at the top of each graph reflect correlation and consistency of expression data with the cell specific gene signature. For p-values > 0.05, we cross compared with FACS data and found correlation between both platforms. Data with p > 0.05 should be taken as a preliminary guide in the absence of FACS data. For cell types without p-values, only one gene was used to estimate population abundance. Medians of each immune population are indicated as bars. Statistical significance between groups: * 0.01 < p < 0.05, ** 0.001 < p < 0.01, *** p < 0.001. (TIF) [file pone.0206223.s010.tif]

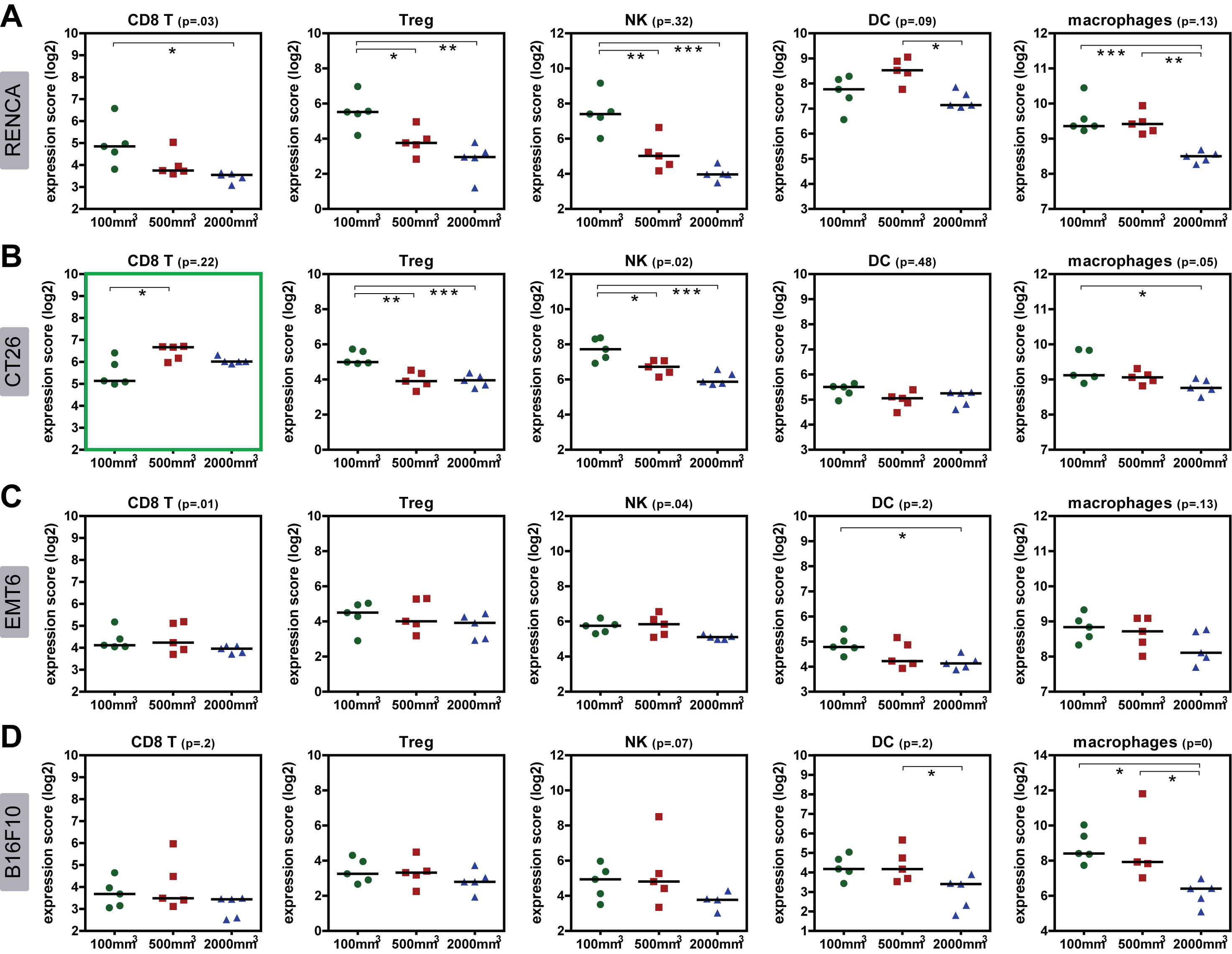

Supplement: S2 Fig — Abundance of immune cell populations was determined by total tumor RNA analysis using the PanCancer Immune profiling panel. Immune populations changes with tumor progression in (A) RENCA, (B) CT26, (C) EMT6, and (D) B16F10. The p-values listed at the top of each graph reflect correlation and consistency of expression data with the cell specific gene signature. Data with p > 0.05 should be taken as a preliminary guide in the absence of FACS data. For cell types without p-values, only one gene was used to estimate population abundance. The green box highlights CD8 T cell increase with tumor volume increase in the CT26 model, which is consistent with FACS data. Medians of each immune population are indicated as bars. Statistical significance between groups: * 0.01 < p < 0.05, ** 0.001 < p < 0.01, *** p < 0.001. (TIF) [file pone.0206223.s011.tif]

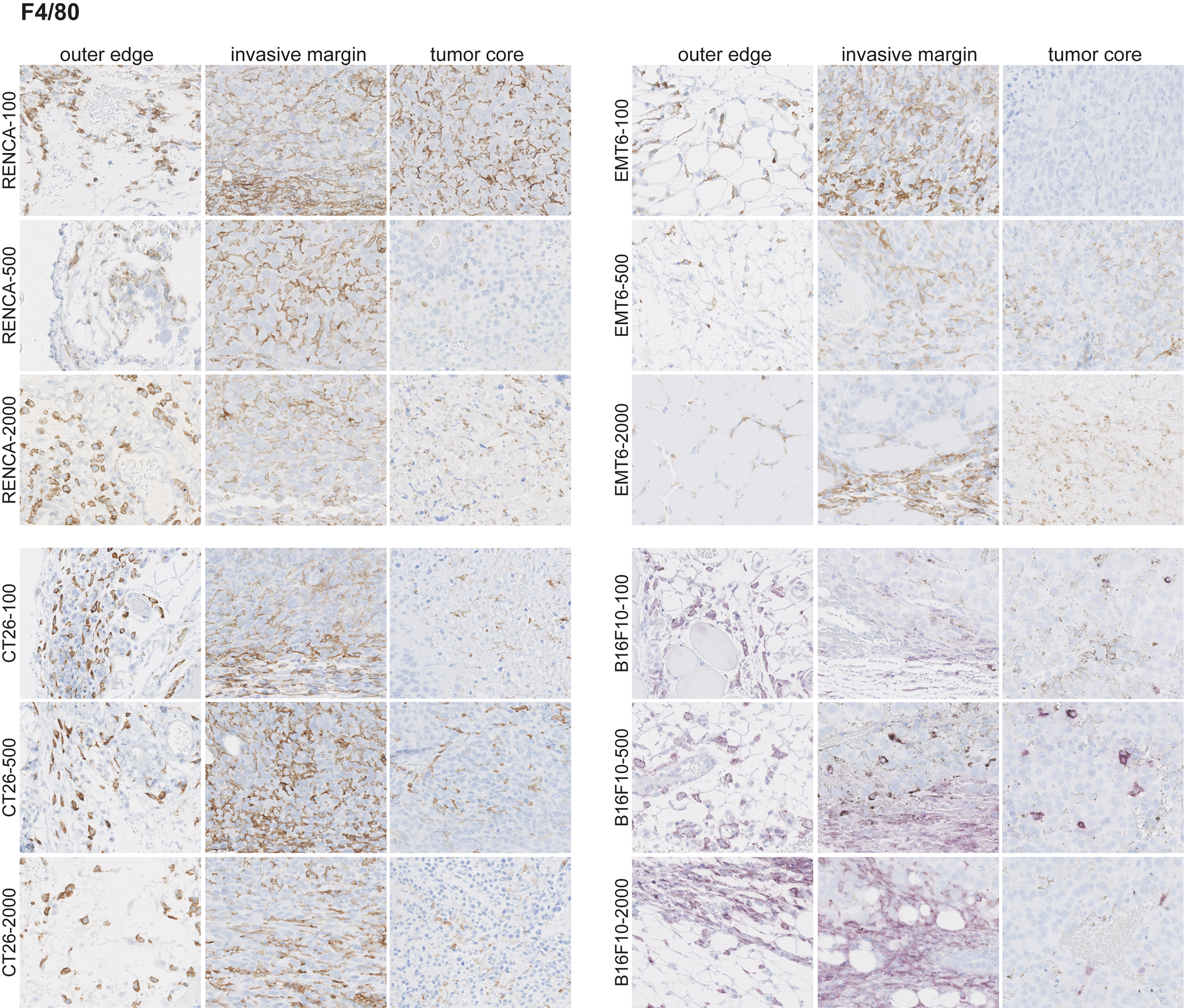

Supplement: S3 Fig — IHC was performed on fixed and paraffin embedded tumor samples across the different models and across all tumor sizes. Five mice per model at each tumor size were used for this analysis. A representative image for each is shown. (TIF) [file pone.0206223.s012.tif]

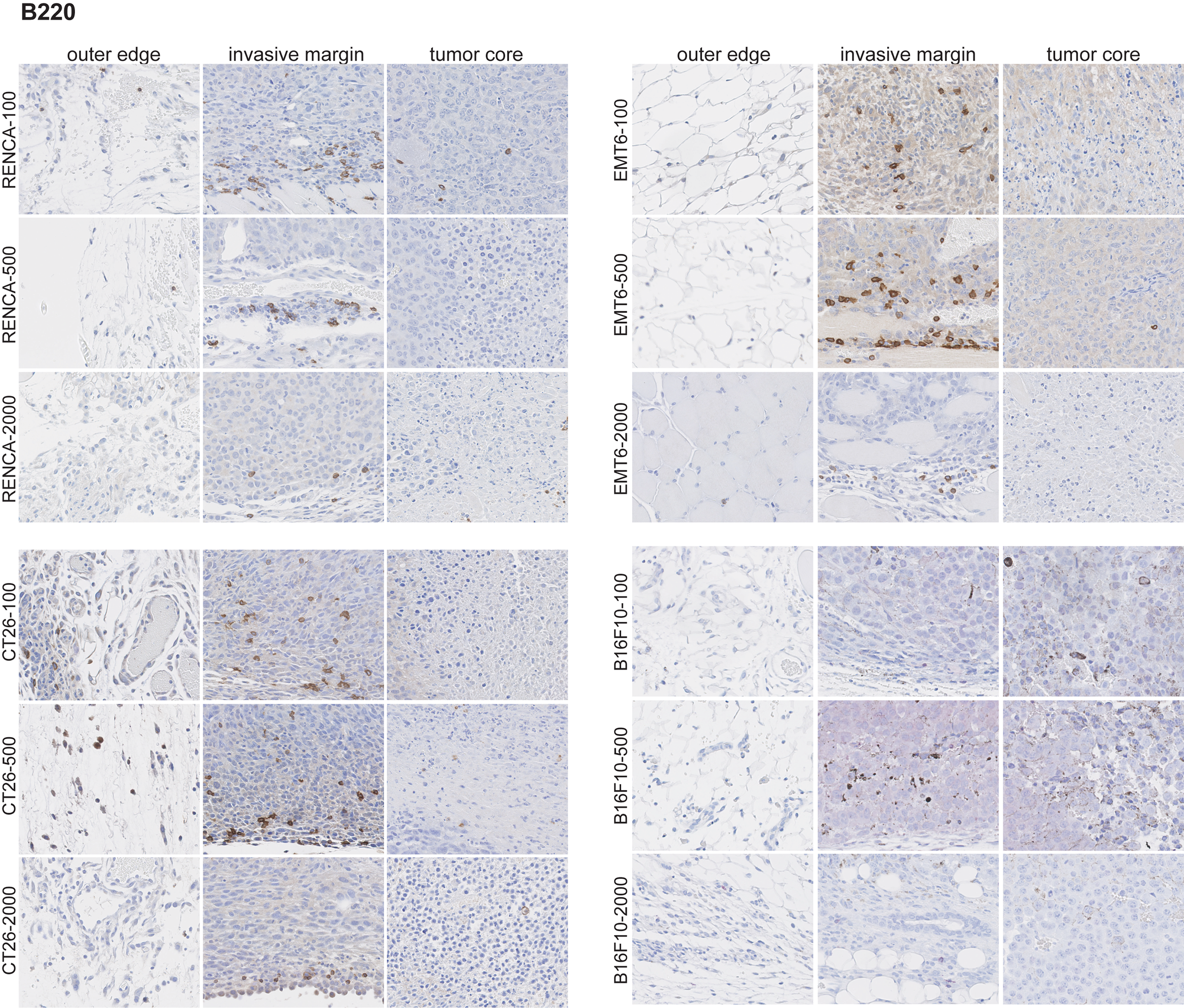

Supplement: S4 Fig — IHC was performed on fixed and paraffin embedded tumor samples across the different models and across all tumor sizes. Five mice per model at each tumor size were used for this analysis. A representative image for each is shown. (TIF) [file pone.0206223.s013.tif]
